# ﻿First record of *Orthohalarachneattenuata* in *Arctocephalusaustralis* in mainland Argentina (Parasitiformes, Mesostigmata, Dermanyssoidea, Halarachnidae) with observations on its ambulacral morphology

**DOI:** 10.3897/zookeys.1207.127297

**Published:** 2024-07-24

**Authors:** Andrés Osvaldo Porta, Juan Pablo Loureiro, Marcela Karina Castelo

**Affiliations:** 1 División de Aracnología, Museo Argentino de Ciencias Naturales Bernardino Rivadavia, Av. Ángel Gallardo 470 C1405DJR, Buenos Aires, Argentina; 2 Universidad de Buenos Aires, Facultad de Ciencias Exactas y Naturales, Departamento de Ecología, Genética y Evolución, Instituto de Ecología, Genética y Evolución de Buenos Aires (IEGEBA, UBA-CONICET), Pabellón II, Ciudad Universitaria, Buenos Aires C1428EGA, Argentina; 3 Departamento de Ciencias Exactas, Universidad Nacional del Oeste, Belgrano 369 C1718, San Antonio de Padua, Buenos Aires, Argentina; 4 Fundación Mundo Marino, Av. X 157, San Clemente del Tuyú B7105, Provincia de Buenos Aires, Argentina; 5 Laboratorio de Entomología Experimental – Grupo de Investigación en Ecología de los Mares (LEE-GIEM), Instituto de Ecología, Genética y Evolución de Buenos Aires (IEGEBA-CONICET/UBA), Departamento de Ecología, Genética y Evolución, Facultad de Ciencias Exactas y Naturales, Universidad de Buenos Aires, Pabellón II, Ciudad Universitaria, Buenos Aires C1428EGA, Argentina

**Keywords:** Acari, attachment structures, marine mammals, mites, Otariidae, parasites

## Abstract

Pinniped respiratory mites of the species *Orthohalarachneattenuata* have been recorded from various locations around the world but not from continental Argentina. In the present work, we document for the first time the presence of *O.attenuata* on *Arctocephalusaustralis* on the Argentine mainland. A total of 23 adult and 381 immature mites were collected from the nose and nasopharyngeal cavity during a necropsy. The mite ambulacrum is described in adults and larvae. This structure consists of a pretarsus, an extensible pulvillum, a pair of claws and paradactyli (pretarsus opercula). The ambulacral structures also have some peculiarities, such as the presence of longitudinal furrows in the claws, straight claws in legs II and III (as opposed to curved in legs I and IV), and the fin-shaped paradactyli. The morphology of the ambulacrum of this mite is interpreted as an adaptation for anchoring to different surfaces of the host, and the protective structures present in the larvae as an adaptation for the dispersal phase in the external environment.

## ﻿Introduction

Mites of the family Halarachnidae Oudemans, 1906 (Acari, Mesostigmata) are obligate parasites of the respiratory tract of a variety of mammals (Lindquist et al. 2009) and are usually found in the mucosa of the nasal cavity, upper respiratory tract, and lungs. Species in the genus *Orthohalarachne* affect otariids (sea lions and fur seals) and odobenids (walruses). Two species of *Orthohalarachne* have been described that affect marine mammals: *O.diminuata* (Doetschman, 1944) and *O.attenuata* (Banks, 1910).

*Orthohalarachneattenuata* has been reported as a parasite of several species of pinnipeds in various parts of the world, but not from mainland Argentina (Table 1). Adults and larvae are found in the upper respiratory tract of their hosts (Kim et al. 1980). Newell (1947) reported that *Orthohalarachne* species are found in the North Atlantic, the Pacific coast of North America, and the Islas Malvinas (Falkland Islands).

**Table 1.** Otarid and odobenid species documented as hosts of *Orthohalarachneattenuata* mites in different parts of the world.

Host species	Common name	Reference(s)	Geographic area(s)
*Arctocephalusaustralis* (Zimmermann, 1783)	South American sea lion	Katz et al. 2012; Gastal et al. 2016; Seguel et al. 2018; Duarte-Benvenuto et al. 2022	Cabo Polonio, Isla de Lobos (Uruguay); Rio Grande do Sul, San Pablo state (Brazil); Punta San Juan (Peru)
*Arctocephalusphilippiitownsendi* Merriam, 1897	Guadalupe fur seal	Pesapane et al. 2021	Central California coast (USA)
*Arctocephaluspusillusdoriferus* Wood Jones, 1925	Australian fur seal	Domrow 1963, 1974; Seawright 1964; Tubb 1937	Port Lincoln, Lady Julia Percy Island, Portarlington, Dangerous Reef, Seal Rocks and Geelong (Australia); New South Wales coast (England)
*Callorhinusursinus* Linnaeus, 1758	Northern fur seal	Dunlap et al. 1976; Kim et al. 1980; Kikuchi and Okuyama 1987; Kuzmina et al. 2021; Pesapane et al. 2021	Pribilof Islands, St. Paul Island, Alaska (USA). Hokkaido (Japan); Central California coast (USA)
*Eumetopiasjubatus* (Schreber, 1776)	Steller sea lion	Fay and Furman 1982; Konishi and Shimazaki 1998	Alaska (USA); Hokkaido (Japan)
*Neophocacinerea* Peron, 1816	Australian sea lion	Domrow 1974; Marlow 1975; Nicholson and Fanning 1981	Port Lincoln, Dangerous Reef, Seal Rocks and Geelong, Kangaroo Island (Australia)
*Odobenusrosmarusdivergens* (Illiger, 1815)	Pacific walrus	Fravel and Procter 2016	Alaska (USA)
*Otariaflavescens* Shaw, 1800	Southern sea lion	Finnegan 1934; Gomez-Puerta and Gonzales-Viera 2015; Seguel et al. 2018; Rivera-Luna et al. 2023	Islas Malvinas (Argentina); Lima, Punta San Juan (Peru); Valdivia (Chile)
*Zalophuscalifornianus* (Lesson, 1828)	California sea lion	Pesapane et al. 2021, 2022	Central California coast (USA)
*Zalophuswollebaeki* Sivertsen, 1953	Galapagos fur seal	Kuzmina et al. 2018; Pesapane et al. 2021	Central California coast (USA)

In terms of mite development, the larva of *O.attenuata* is an active stage that attaches to nasal structures. It can survive for several days without feeding and, after dispersal among hosts, is followed by two short or suppressed nymphal stages that do not feed and are generally not observed (Furman and Smith 1973). The adult is an active feeding stage, with individuals typically attaching themselves to tissues via tarsal structures that pierce the respiratory epithelium with chelicerae and feed on lymph and other body fluids (Dowling 2006). Females of *O.attenuata* are up to 4 mm long due to their elongated opisthosoma (Banks 1910). Newell (1947) undertook a systematic revision of the halarachnid mites parasitising Pinnipedia by observing morphological characters with light microscopy and established the genus *Orthohalarachne*. With advances in microscopy techniques, it is now possible to analyse morphological characters that are difficult to observe by light microscopy alone. In this sense, the specific adaptations of the host-tissue anchoring structures have not been described in detail.

In this article, we report for the first time the presence of *O.attenuata* in continental Argentina parasitizing *A.australis*, describe in detail the morphology of the ambulacrum in adults and larvae using SEM techniques, and interpret these morphological features in terms of the mechanism of attachment of the mites to host tissues. Finally, we discuss the taxonomic status of *Orthohalarachne* mites in the context of previous descriptions.

## ﻿Materials and methods

### ﻿Methods of collection

**Figure 1.**
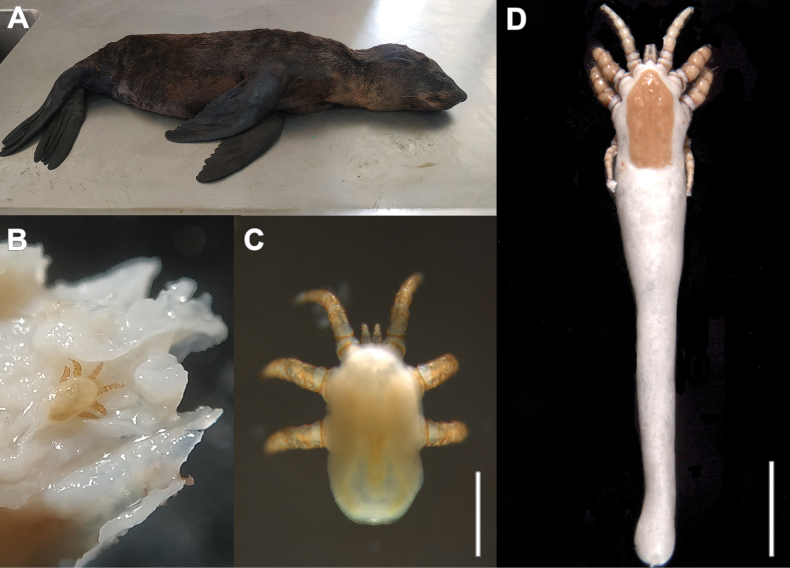
Marine mammal host and mites collected **A** young male of *Arctocephalusaustralis* (ID M7422), host of the collected mites **B***Orthohalarachneattenuata*, larva (LEE-FCEN-UBA), in the turbinate tissue of the host **C***O.attenuata*, larva (LEE-FCEN-UBA), dorsal habitus **D***O.attenuata*, female (LEE-FCEN-UBA), dorsal habitus. Scale bars: 0.5 mm (**C**); 1 mm (**D**).

Mites were collected from a South American fur seal, *Arctocephalusaustralis* (Zimmermann, 1783) (Carnivora, Otariidae) (Fig. 1A), rescued from the beaches of Las Toninas, Province of Buenos Aires, Argentina (36°29′00″S, 56°42′00″W) in August 2022. The animal was transferred to the Mundo Marino Foundation’s Rescue and Rehabilitation Centre, where it was assisted in its recovery (ID M7422, young male). When the specimen did not recover and died, a necropsy was performed, and the entire respiratory system was removed. The sea lion’s respiratory organs were then stored in the freezer, dissected, and washed for manual collection of mites. During the dissection of the respiratory organs, the nose, turbinates, nasopharynx, pharynx, trachea, and lungs were separated. All mites present in the respiratory tissues were then collected manually with forceps. The specimens collected in this way were counted and grouped by organ into larvae or adults and fixed in 96% alcohol in a freezer until used for taxonomic studies.

### ﻿Specimen handling and imaging

Specimens for optical observation were mounted in Hoyer’s medium following Walter and Krantz (2009). Measurements were made using an Olympus CH or Leica D2500 compound microscope. For scanning electron microscopy, specimens were dehydrated according to Pérez-Benavides et al. (2023). The specimens were processed in amyl acetate, mounted with copper adhesive tape, sputter-coated with gold-palladium (60:40) and examined with a ZEISS GeminiSEM 360.

Optical images of preserved specimens were taken using a Leica DFC 290 digital camera mounted on a Leica M165 C stereomicroscope in multiple focal planes, with focal planes aligned using Helicon Focus 4.62.2.

For the structural description of the mites, the morphological terminology follows Walter and Krantz (2009) and for the ambulacrum Alberti and Coons (1999). The studied material is housed in the Colección de Artrópodos of the Laboratorio de Entomología Experimental, Facultad de Ciencias Exactas y Naturales, Universidad de Buenos Aires (LEE-FCEN-UBA) and in the Arachnological National Collection, Museo Argentino de Ciencias Naturales Bernardino Rivadavia, in Buenos Aires, Argentina (MACN-Ar 46561 and 46562).

## ﻿Results

**Figure 2.**
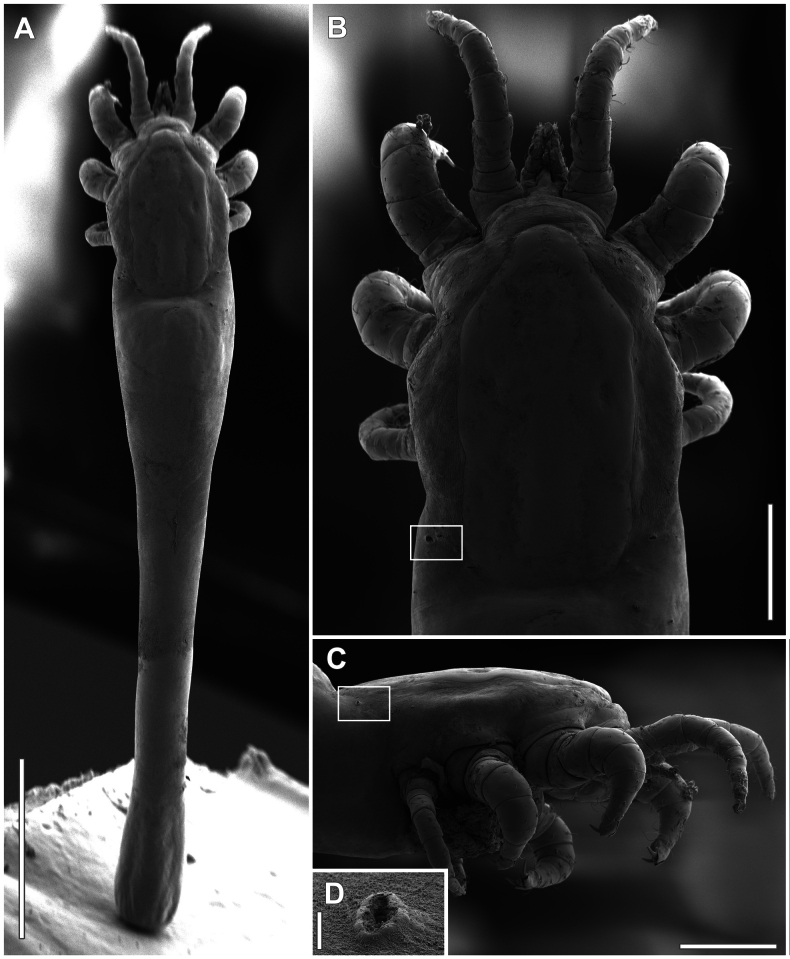
*Orthohalarachneattenuata*, females (MACN-Ar 46561) **A** dorsal habitus **B** same as in **A**, detail of anterior part, white box marks left stigma **C** lateral view of anterior part of body, white box marks left stigma **D** detail of the stigma. Scale bars: 1 mm (**A**); 300 µm (**B, C**); 10 µm (**D**).

We collected 381 larvae (Figs 1B, C, 5A, B) and 23 adults (Figs 1D, 2) from the nose and nasopharynx of *A.australis*. Based on leg and palpal chaetotaxy, idiosoma dimensions, body chaetotaxy, and dorsal sclerites, identity was assigned to *O.attenuata*.

**Figure 3.**
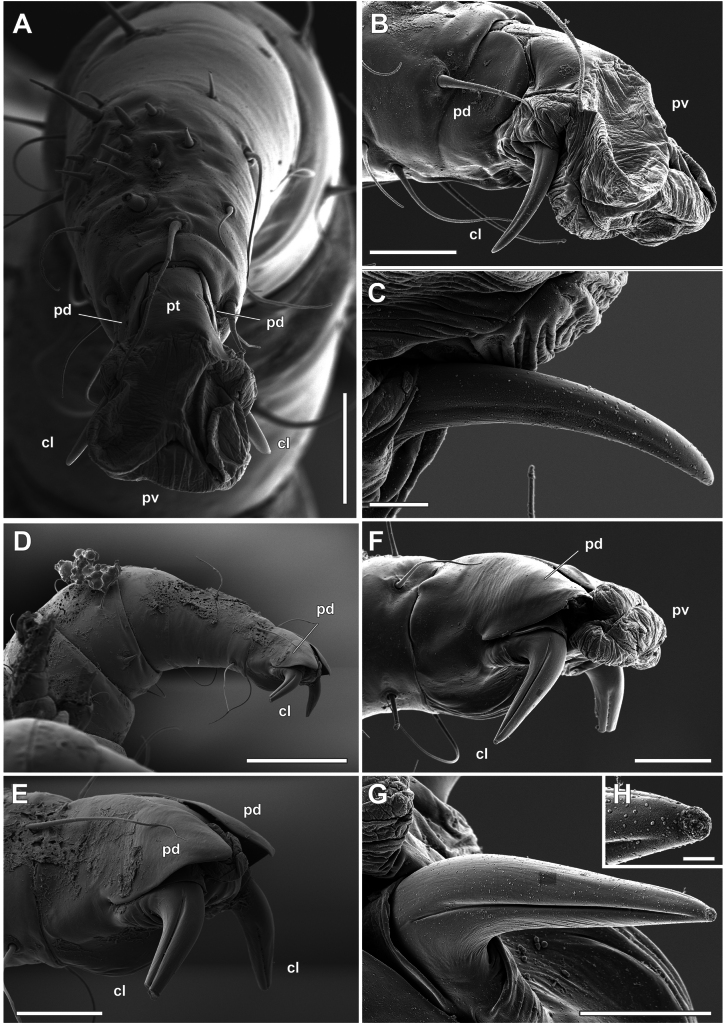
*Orthohalarachneattenuata*, females (MACN-Ar 46561) **A** tarsus and ambulacrum of right leg I, dorsodistal view **B** same as in **A**, detail of the ambulacrum, antiaxial view **C** same as in **B**, detail of the antiaxial claw; antiaxial view **D** tarsus and ambulacrum of left leg II, paraxial view **E** as in **D**, detail of the ambulacrum **F** ambulacrum of right leg II, antiaxial view **G** as in **F**, detail of the antiaxial claw; antiaxial view **H** as in **G**, detail of the distal part. Abbreviations: *cl*, claw; *pd*, paradactyl; *pt*, pretarsus; *pv*, pulvillus. Scale bars: 30 µm (**A**); 20 µm (**B, G**); 5 µm (**C**); 100 µm (**D**); 30 µm (**E, F**); 2 µm (**H**).

**Figure 4.**
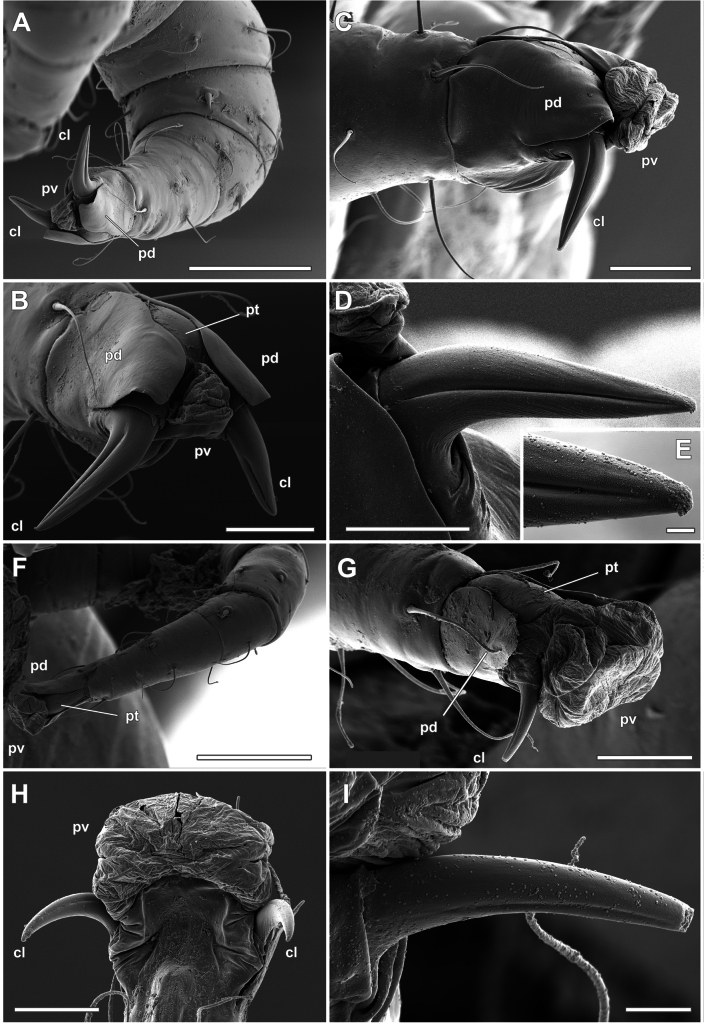
*Orthohalarachneattenuata*, females (MACN-Ar 46561) **A** right leg III, paraxial view **B** Same as in **A**, ambulacrum **C** ambulacrum of right leg III, antiaxial view **D** same as in **C**, detail of the antiaxial claw antiaxial view **E** as in **D**, detail of the distal part **F** tarsus and ambulacrum of left leg IV, dorsal view **G** ambulacrum of right leg IV, antiaxial view **H** ambulacrum of left leg IV, ventral view **I** same as in **G**, antiaxial claw. Scale bars: 100 µm (**A**); 30 µm (**B, C**); 20 µm (**D**); 2 µm (**E**); 100 µm (**F**); 30 µm (**G**); 20 µm (**H**); 5 µm (**I**).

The ambulacrum of adult females (Figs 3, 4) is composed of a pretarsus (*pt*, Figs 3A, 4G), a pair of large paradactyli or pretarsal opercula (*pd*), a pair of claws (*cl*), and a large and retractable pulvillus (*pv*) (Figs 3, 4). The paradactyli are large and fin-shaped (Fig. 3A, B, D–F) and do not have any denticles in the distal part. When the pulvillus is retracted, they completely cover the paradactyli (Figs 3D, E, 4A, B). The clearing for optical observation makes these structures difficult to observe. The claws are directed towards the ventral side of the leg and have different shapes and sizes depending on the leg. Legs II and III have relatively large and straight claws (Figs 3G, 4D) with deep longitudinal furrows on both sides of the structure. These furrows do not extend to the distal end (Figs 3H, 4E).

**Figure 5.**
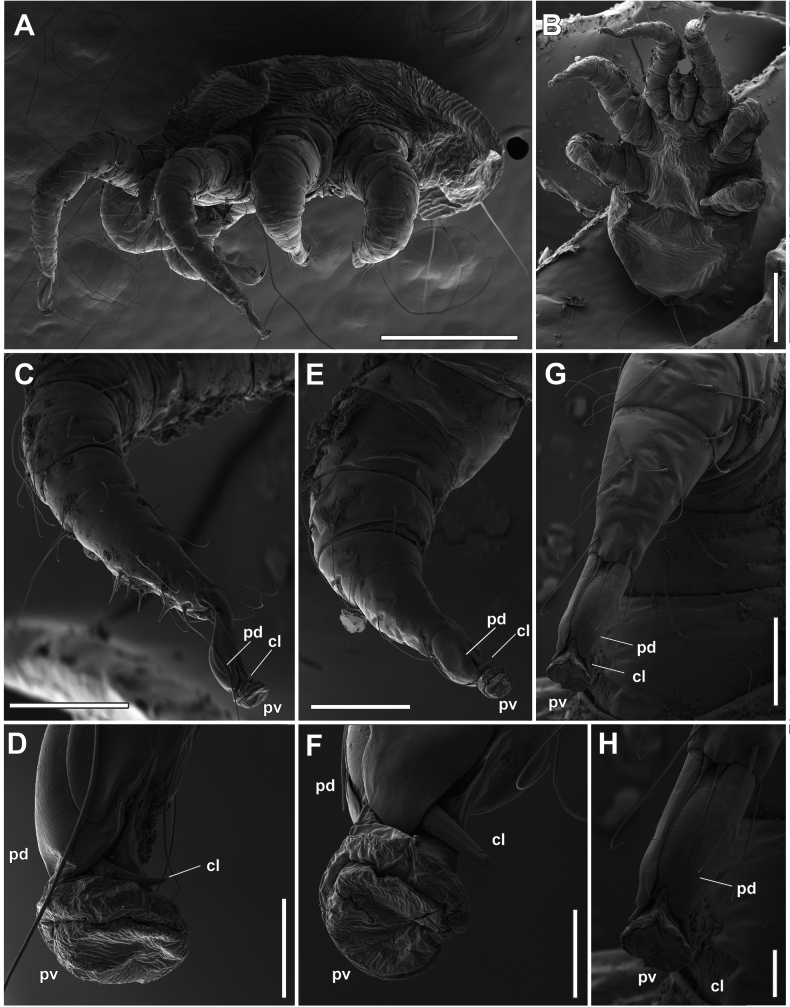
*Orthohalarachneattenuata*, larvae (MACN-Ar 46561) **A** habitus, lateral view **B** habitus ventral view **C** right leg I, paraxial view **D** as in **C**, ambulacrum **E** right leg II, paraxial view **F** as in **E**, ambulacrum **G** right leg III, dorsal view **H** as in **G**, ambulacrum. Scale bars: 300 µm (**A, B**); 100 µm (**C**); 20 µm (**D**); 100 µm (**E**); 20 µm (**F**); 60 µm (**G**); 20 µm (**H**).

In contrast, the claws on legs I (Fig. 3B, C) and IV (Fig. 4F–I) are more curved and relatively smaller, with longitudinal furrows much shallower than in legs II and III. The pulvilli are smooth on all legs (Figs 3B, F, 4C, G, H) and, when expanded (*cfr*, Figs 3B, F, 4C, G), are rather large and directed anteriorly to the longitudinal axis of the tarsi. In larvae (Fig. 5) the ambulacrum is composed of the same structures as in adults, but the paradactyli are more elongated in the longitudinal axis (Fig. 5C, E, G), and cover most of the pretarsus and the claws when the pulvilli are expanded (Fig. 5D, F, H).

## ﻿Discussion

In this work, after an exhaustive taxonomic determination, we document for the first time the presence of *Orthohalarachneattenuata* parasitizing *A.australis* in continental Argentina, extending its known geographical distribution. Furthermore, we describe in detail the structure of the ambulacrum through SEM images of the adult and larvae, observing structural differences and details of its anchoring apparatus to the host that have never been described in such detail, reflecting the different function they have in locomotion at each stage of the life cycle.

Detailed studies describing the ambulacral morphology of dermanyssoid mites are scarce (Evans and Till 1965; Evans 1992; Alberti and Coons 1999; Montasser 2006; Krantz 2009; Di Palma and Mul 2019). In these mites (as free-living Gamasida), the ambulacrum consists of a pretarsus, a pair of claws, a pulvillus and the paradactyli (Alberti and Coons 1999). In general, the paradactyli (pretarsal opercula) may be variable or absent on the first pair of legs, with their distal end generally dentate (Evans and Till 1979: fig. 14G). In some taxa, however, paradactyli can have different shapes with modifications in orientation depending on the life history of the mite (e.g. Pugh et al. 1987). In *O.attenuata*, the shape of these structures differs in adults and larvae, probably fulfilling different functions depending on the life history of each stage. While larvae have elongated paradactyli that seem to cover the entire ambulacrum, even the claws (Fig. 5C–H), in adults they only protect the pulvillus when it is not expanded (*cfr*, Fig. 3D, E). The peculiar morphology of the paradactyli on the larval legs could be related to their high dispersal in the environment (Furman and Smith 1973). In this process, mite larvae are expelled from the infected host’s nose by sneezing, fall onto the substrate or onto the body of another host. The larvae must then crawl on their legs along hot, hard, or rough surfaces until they find the nostrils of a new host (Furman and Smith 1973). Therefore, we propose that paradactyli play an important protective role in locomotion during the host-finding process on the beach. In contrast, we observed that adult mites do not have tarsi with protective structures. During development, nymphs moult within the host’s respiratory tract and adults remain immobile, mainly in the most internal respiratory organs such as the nasopharynx, do not leave the host and remain attached to the internal mucosa for the rest of their lives. We propose that adults have tarsi with fewer protective structures as an adaptation to their reduced locomotor activity. Unfortunately, we did not collect nymphs of this species, but considering the differential development of tarsal claws in these stages (Furman 1977), it would be very interesting to study the development of these structures in nymphal stages using SEM techniques in a future work. However, it has been reported that it is very difficult to find halarachnid nymphs in otariids due to the extremely short duration of the protonymphal and deutonymphal stages, an adaptation in these mites to their highly specialised parasitic lifestyle (Furman and Smith 1973).

We interpret the presence of longitudinal furrows in the claws of these mites as an adaptation for attachment to the host’s respiratory mucosa, combined with the presence of straight claws on legs II and III, shaped like the head of a climbing axe, for attachment to a soft substrate. On the one hand, it is noteworthy that these claws appear to be firmly inserted into the turbinates and the mucosa of the nasal cavity. In fact, removal of the material results in breakage of the distal portion of the claw. On the other hand, the presence of a large, retractable, and smooth pulvillus is a common adaptation in dermanyssoid mites to adhere to a smooth surface, which would correspond to the anchoring of adults in the mucosa of the choanae and of larvae in the mucosa and hard tissue of the turbinates. Therefore, we propose that both structures, claw and pulvillus, may act alternatively as attachment devices depending on the substrate to which the mite is attached, as similar attachment structures have been recorded for other mites such as *Dermanyssusgallinae* (De Geer, 1778) (Di Palma and Mul 2019). When collecting mites from different host tissues, we observed that almost all adults were attached to the soft mucosa of the choanae, whereas larvae were mostly found in harder tissues such as the nose and turbinates. The differences in the structure of the ambulacrum of each stage would then correspond to the hardness characteristics of the host tissues in which the individuals of each stage were found. According to the life cycle of this mite, the characteristics of the tarsal structures found in larvae and adults in this work correspond to adaptations to parasitic life in organisms that have to alternate between being inside and outside the host depending on the stage of development.

With regard to the taxonomic status of *Orthohalarachne* mite species in the literature, a description of a mite species on another host with similar characteristics to *O.attenuata* is reported. The halarachnid mite *O.magellanica* (Finnegan, 1934) was described on *O.flavescens* Shaw, 1800 from the Islas Malvinas (Falkland Islands), but this description does not mention the leg chaetotaxy. In the revision of the family, Newell (1947) treated this species as valid and mentioned the relatively shorter dorsal shield (L/W = 1.75–1.8 vs 2.00–2.28 in *O.attenuata*) and the greatly enlarged male trochanter (Finnegan 1934: fig. 11) as diagnostic characters. Later, Domrow (1974: 20) subjectively synonymised this species with *O.attenuata* because “I see no real evidence in the original descriptions to justify the retention of the nominal taxa now combined under *H. attenuata*”. In papers dealing with South American records of *O.attenuata* (e.g. Gomez-Puerta and Gonzales-Viera 2015; Gastal et al. 2016; Ebmer et al. 2022; Rivera-Luna et al. 2023), *O.magellanica* is treated as a junior synonym of *O.attenuata*. In our specimens, the chaetotaxy, at least in number and arrangement, is similar to that reported by Furman (1977) for *O.attenuata*. The L/W ratio of the dorsal shield varies between 2.11 and 2.2 in non-compressed material, while in compressed (MACN-Ar 46562, Hoyer’s mounted) specimens this ratio varies between 1.73 and 2.00. This difference could be the reason for the different observations in the original description of *O.magellanica*. However, considering the stability of the leg chaetotaxy of halarachnid mites (Furman 1977) and the wide geographical distribution of the host species of *O.attenuata*, we believe that Domrow’s synonym remains to be tested using molecular data.

## ﻿Additional information

### Author ORCIDs

Andrés Osvaldo Porta https://orcid.org/0000-0002-9533-4139

Marcela Karina Castelo https://orcid.org/0000-0001-7107-6078

### Data availability

All of the data that support the findings of this study are available in the main text.
